# Occurrence of bladder metastasis 10 years after surgical removal of a primary gastric cancer: a case report and review of the literature

**DOI:** 10.1186/1752-1947-7-204

**Published:** 2013-08-14

**Authors:** Csilla András, László Tóth, János Pósán, Emese Csiki, Miklós Tanyi, Zoltán Csiki, Zoltán Garami, Attila Enyedi, Tibor Flaskó, Zsolt Horváth

**Affiliations:** 1 Institute of Oncology, Medical and Health Science Center, University of Debrecen, Nagyerdei krt. 98, 4032 Debrecen, Hungary; 2 Department of Pathology, Medical and Health Science Center, University of Debrecen, Nagyerdei krt. 98, 4032 Debrecen, Hungary; 3 Department of Surgery, Medical and Health Science Center, University of Debrecen, Nagyerdei krt. 98, 4032 Debrecen, Hungary; 4 Institute of Internal Medicine, Medical and Health Science Center, University of Debrecen, Nagyerdei krt. 98, 4032 Debrecen, Hungary; 5 Department of Urology, Medical and Health Science Center, University of Debrecen, Nagyerdei krt. 98, 4032 Debrecen, Hungary

**Keywords:** Gastric tumor, Bladder adenocarcinoma, Secondary bladder cancer, Secondary peritoneal metastasis

## Abstract

**Introduction:**

Secondary bladder neoplasms are uncommon and they represent only 2% of all malignant bladder tumors.

**Case presentation:**

The authors present a case of a 59-year-old Caucasian man with a primary gastric adenocarcinoma that had been surgically removed 10 years before he developed bladder metastasis. He presented with low abdominal pain after 10 years without any symptoms. Cystoscopy and an abdominal computed tomography scan showed a bladder tumor. A transurethral resection of the bladder tumor was performed. A histological examination revealed an adenocarcinoma, which turned out to be a metastasis of the primary gastric tumor. One year later, abdominal surgery revealed peritoneal metastases.

**Conclusion:**

This is the first known case in Europe where bladder metastasis occurred 10 years after surgical removal of a primary gastric neoplasm. There are only four cases in the literature where metastases of the peritoneum developed 11 years after surgical removal of a primary gastric tumor.

## Introduction

Bladder tumor is the eighth most common neoplasm in Hungary (the fifteenth most common in women and the sixth most common in men) [1]. More than 95% of primary bladder tumors are transition cell carcinomas, while approximately 1% is adenocarcinoma. Secondary bladder neoplasms are uncommon and they represent only 2% of all malignant bladder tumors. Secondary bladder neoplasms usually arise from a primary colon, prostatic, or cervical tumor or from organs surrounding the bladder itself. Metastases usually originate from gastric or breast neoplasms, and melanoma. Krukenberg metastasis of a gastric tumor in women (an ovarian metastasis from a gastric neoplasm) could be a direct source of a secondary bladder malignancy. Symptoms appear in approximately 20% of metastatic cases, while the majority of cases remain asymptomatic and only autopsy reveals vesical involvement. As primary adenocarcinoma of the bladder is rare, when a tumor is found, it should alert the clinician for a possible metastatic origin; therefore, further investigations should be carried out to find the primary tumor. In publications where bladder metastasis originated from a primary gastric neoplasm, secondary malignancy occurred within two years after the diagnosis of the primary tumor. Most of these cases were reported from Japan [2-9]. Only a few cases were published where a secondary tumor occurred 10 years after the surgical removal of a primary gastric neoplasm [10]. Gastric neoplasms usually metastasize into the liver, lung, abdominal lymph nodes, bones and the peritoneum. Bladder metastasis usually occurs after the primary tumor has become widespread with peritoneal metastases. A solitary bladder metastasis is extremely rare.

We report a single case in a man with late urinary bladder metastasis 10 years after the surgical removal of the primary gastric adenocarcinoma.

## Case presentation

**Figure 1:**
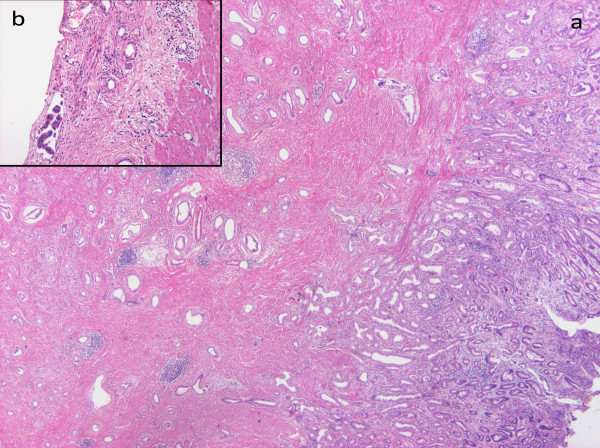
**(a) Histopathology of the stomach representing adenocarcinoma of the stomach involving all the layers of the stomach wall (hematoxylin and eosin ×20). (b)** tumor cells in the subserous lymphoid vessels (hematoxylin and eosin ×200).

**Figure 2:**
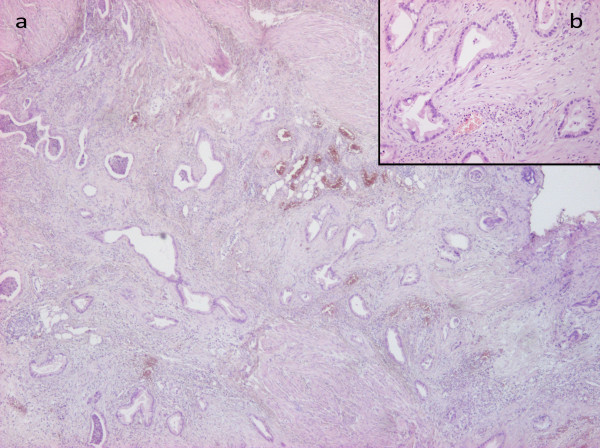
**(a) Bladder biopsy specimen showing well-differentiated tubular adenocarcinoma (hematoxylin and eosin ×40). (b)** with higher magnification (hematoxylin and eosin ×200).

A 59-year-old Caucasian man presented to his doctor with gastric complaints. Gastroscopy showed an ulcer with a diameter of 5cm along the lesser curvature in the prepyloric antrum. A biopsy revealed adenocarcinoma of the stomach. The patient underwent a Billroth II resection of the stomach, omentectomy and D2 lymph node dissection. An anatomical preparation showed an exulcerated and infiltrative tumor invading all layers of the stomach (Figure 1). Histopathologic evaluation revealed well-differentiated exulcerated adenocarcinoma with highly desmoplastic stroma and malignant cells arranged in solid nests and glands with mucin production at TNM stage III, pT3, pN2. Eight out of 18 removed perigastric lymph nodes showed metastatic foci. Lymphatic vessel involvement was observed. According to Lauren's classification, this tumor was an intestinal type with low proliferative fraction, in World Health Organization (WHO) classification, it was tubular adenocarcinoma. Only 2 to 3% of tumor cells were molecular immunology Borstel-1 (MIB1)+, which justified its slow runoff. Abdominal and chest computed tomography (CT) scans did not show distant metastasis. The patient was treated with six cycles of cisplatin and VePesid® (etoposide) chemotherapy after surgery. We did not observe any sign of local recurrence or distant metastasis during follow-up. Our patient was admitted again with low abdominal pain and discomfort 10 years after the gastric tumor diagnosis. Ultrasound showed a 2x4cm unidentified mass on the left posterior wall of the bladder. A CT scan detected a tumor-like mass on the left side of the bladder. Cystoscopy revealed mucosal hyperemia and a tumor close to the left ureteric orifice. We did not detect any other malignancies neither in the thorax, nor in other parts of the abdomen. A transurethral resection of the left-sided bladder tumor was performed. A pathologic examination described mucosal inflammation, intact epithelium, local diffuse muscle invasion (especially in the outer muscular layers), a connective tissue-rich stroma and atypical glandular proliferation with atypical simple columnar-cuboidal epithelium (Figure 2).

**Figure 3:**
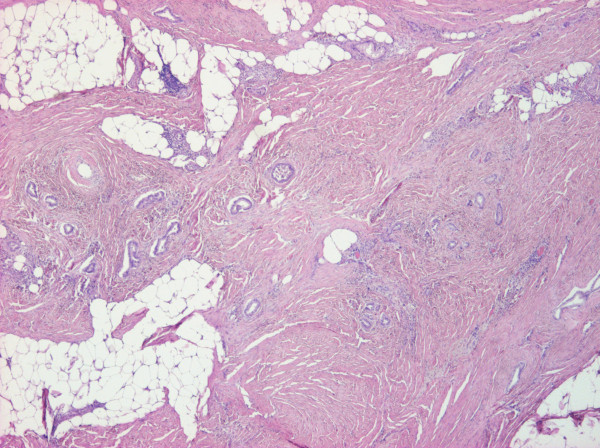
Histopathology of the peritoneum, presenting adenocarcinoma (hematoxylin and eosin ×200).

Immunohistochemical staining of tumor cells detected epithelial membrane antigen (EMA)+, cytokeratin (CK)7+, CA19.9 +, CK20-, carcinoembryonic antigen (CEA)+, while prostate-specific antigen (PSA) was irrelevant. Because of its EMA positivity and CK20 negativity this urinary bladder tumor turned out to be secondary to a gastric neoplasm. Endoscopic control of the primary gastric tumor was performed after a pathologic examination, and a biopsy from the scar showed no sign of local recurrence. Positron emission tomography - computed tomography (PET-CT) presented residual malignancy in the bladder. Cystoscopy showed mucosal hyperemia and edema on the left lower part of the posterior wall and necrosis on its surface. The patient received five cycles of epirubicin, cisplatin and capecitabine (ECX) chemotherapy. He responded well to treatment and control PET and CT scans were negative. He became asymptomatic. One year later, a colonoscopy detected a 4cm sessile polyp in the ascending colon. A histopathologic evaluation revealed adenoma with high-grade dysplasia. An exploratory laparotomy was performed and, surprisingly, peritoneal dissemination was found. A histopathological examination proved metastases of the primary gastric neoplasm for a second time (Figure 3). The tumor was CK7+ and CEA+, CK20-, CA19.9 +, caudal-type homeobox transcription factor (CDX)-2-, thyroid transcription factor (TTF)-1-. The surgical specimens were formalin-fixed. Paraffin-embedded sections (5μm) were used for conventional light microscopy. Silane-coated glass slides and 4μm thick sections were used for immunohistochemistry with antigen retrieval. The following commercially available antibodies were used, according to the manufacturers’ recommendations.

Antibodies: CK7: (Dako, Glostrup, Denmark, OV-TL-12/30, 1:100), Ki67: (Dako, MIB-1, 1:100), EMA: (Dako, E29, 1:100), CEA: (Dako, II-7, 1:50), CK20: (Dako, KS20.8; 1:50), CDX-2: (Novocastra, Leica Microsystems, Wetzlar, Germany, AMT28, 1:50), CA19.9: (Novocastra, Leica Microsystems, Wetzlar, Germany, C241, 1:100), TTF-1: (Dako, 8G7G3/1, 1:50), PSA: (Dako, polyclonal, 1:1000).

## Discussion

Bladder metastases are rare and represent only 2% of all bladder tumors. Metastases to urinary bladder could not only originate from the direct spread of a primary tumor, but also from implantation of detached pelvic/ureteric neoplastic cells, lymphogenic/hematogenous or even peritoneal dissemination from a distant primary neoplasm. Gastrointestinal origin is suspected if a biopsy reveals signet-ring cell adenocarcinoma [7,9]. A primary bladder adenocarcinoma is a very rare tumor. Some 282 secondary bladder tumors were analyzed at Royal Marsden Hospital, which represented only 2.3% of all bladder neoplasms examined by them. The localizations of the primary tumor were the following: 21% originated from the colon, 19% had a prostatic origin, 12% originated from the rectum and 11% from the cervix. In the cases mentioned above a direct spread of a primary neoplasm caused a bladder metastasis. In 4.3% of all cases they found a primary gastric carcinoma, 96.7% were solitary metastasis located in the trigone or in the neck of the bladder. A total of 54% of cases were adenocarcinoma [11].

In our case, the metastatic tumor in the bladder occurred 10 years after the primary neoplasm. Surgical intervention was decided, as we could not detect any metastasis elsewhere and could not prove local recurrence in the stomach. The literature suggests chemotherapy in case of multiple metastases. We first diagnosed bladder metastasis and one year later peritoneal metastases. During the redactation of present article the patient was still alive. Peritoneal metastases can be detected in only 17% of gastric carcinomas [12], which could be caused by direct serosal spread, lymphogenic or vascular dissemination. Overall survival after peritoneal metastases is usually less than six months.

There are only four cases in the literature where peritoneal metastases occurred 10 years after resection of a primary neoplasm. Two cases were reported from Japan, two reports from Korea, but none from Europe. In all four cases, serosal surfaces and lymph nodes were also histologically affected. In these cases, peritoneal metastases occurred within five years after surgery but a 10-year interval for metastasis manifestation is very rare [13-15].

## Conclusion

The authors presented a rare case where, 10 years after the surgical removal of a primary gastric neoplasm, first a bladder and a year later peritoneal metastases occurred. This is the first known case in Europe where, more than 10 years after surgical removal of the primary gastric neoplasm, vesical and peritoneal metastases occurred. In a case of bladder adenocarcinoma, one should always think of the possibility of metastasis, even years after surgical removal of a primary neoplasm. In a case of an unknown primary tumor, further investigations are advised in order to find a possible source of the metastasis. As a solitary bladder metastasis of gastric cancer is rare, one should always search for peritoneal involvement as well. In our case, one year after the diagnosis of a bladder metastasis peritoneal metastases were found during abdominal surgery. Imaging techniques showed no sign of peritoneal involvement. All of the three surgical specimens (stomach, bladder, and peritoneum) contained the same histopathologic type of tumor.

With our case presentation, we would like to stress the necessity at routine disease control to search for distant metastases even 10 years after the primary intervention.

## Consent

Written informed consent was obtained from the patient for publication of this case report and any accompanying images. A copy of the written consent is available for review by the Editor-in-Chief of this journal.

## Competing interests

All authors declare that they have no competing interests.

## Authors’ contributions

CA was a major contributor in writing the manuscript. LT performed and interpreted the histological examinations. JP participated in and interpreted the gastric tumor operation. EC was a contributor in writing the manuscript and did the literature search. MT interpreted the surgical anamnesis and performed the imaging examinations. ZC performed the endoscopy, and gastroenterological controls, and interpreted these data. ZG analyzed the data of the surgical controls. AE participated in and interpreted the second operation. TF performed and interpreted the urological controls and examinations. ZH did the final draft proofreading and approval. All authors read and approved the final manuscript.
